# Heterogeneity of serum gelatinases MMP-2 and MMP-9 isoforms and charge variants

**DOI:** 10.1111/jcmm.12181

**Published:** 2013-12-03

**Authors:** Rocco Rossano, Marilena Larocca, Lea Riviello, Maria Gabriella Coniglio, Jennifer Vandooren, Grazia Maria Liuzzi, Ghislain Opdenakker, Paolo Riccio

**Affiliations:** a Department of Sciences, University of BasilicataPotenza, Italy; b Hospital “Madonna delle Grazie”, Centre for Multiple SclerosisMatera, Italy; c Department of Microbiology and Immunology, KU Leuven, Rega Institute for Medical Research, University of LeuvenLeuven, Belgium; d Department of Biosciences, Biotechnology, and Biopharmaceutics, University of BariBari, Italy

**Keywords:** zymography, matrix metalloproteinases, multiple sclerosis

## Abstract

The matrix metalloproteinases (MMPs) gelatinase A (MMP-2) and gelatinase B (MMP-9) are mediators of brain injury in multiple sclerosis (MS) and valuable biomarkers of disease activity. We applied bidimensional zymography (2-DZ) as an extension of classic monodimensional zymography (1-DZ) to analyse the complete pattern of isoforms and post-translational modifications of both MMP-9 and MMP-2 present in the sera of MS patients. The enzymes were separated on the basis of their isoelectric points (pI) and apparent molecular weights (Mw) and identified both by comparison with standard enzyme preparations and by Western blot analysis. Two MMP-2 isoforms, and at least three different isoforms and two different states of organization of MMP-9 (the multimeric MMP-9 and the N-GAL-MMP-9 complex) were observed. In addition, 2-DZ revealed for the first time that all MMP-9 and MMP-2 isoforms actually exist in the form of charge variants: four or five variants in the N-GAL complex, more charge variants in the case of MMP-9; and five to seven charge variants for MMP-2. Charge variants were also observed in recombinant enzymes and, after concentration, also in sera from healthy individuals. Sialylation (MMP-9) and phosphorylation (MMP-2) contributed to molecular heterogeneity. The detection of charge variants of MMP-9 and MMP-2 in MS serum samples illustrates the power of 2-DZ and demonstrates that in previous studies MMP mixtures, rather than single molecules, were analysed. These observations open perspectives for better diagnosis and prognosis of many diseases and need to be critically interpreted when applying other methods for MS and other diseases.

## Introduction

Multiple sclerosis (MS) is a multifactorial, chronic and debilitating disease of the central nervous system (CNS), mostly in young adults 1. Its pathogenesis has all the characteristics of neuroinflammatory and autoimmune diseases, as it involves the attack by autoreactive T cells, B lymphocytes, macrophages and microglial cells against brain white matter 2,3.

The pathological hallmarks of MS lesions are perivascular inflammation, breakdown of the blood–brain barrier (BBB), variable axonal and oligodendrocyte damage, focal demyelination and scar formation 4–6. Myelinotoxic factors include antibodies, activated complement, cytokines, reactive oxygen species (ROS) and matrix metalloproteinases (MMPs).

Matrix metalloproteinases are interesting because they are implicated in a variety of physiological and pathological processes. On one hand, they regulate neurogenesis, oligodendrogenesis and brain plasticity in developing healthy brain. On the other hand, they are involved in neurodegenerative, vascular and inflammatory disorders 7,8. The 25 human MMPs are calcium-activated and Zn^2+^-dependent, extracellular or membrane-bound endopeptidases with different structural and functional domains. Matrix metalloproteinases modulate cell–cell and cell–extracellular matrix (ECM) interactions and appear to have a regulatory role in cell signalling, because they are able to process growth factors, receptors, hormones, adhesion molecules, cytokines and chemokines. This means that the mechanisms by which MMPs influence cell activity in normal or pathological conditions are complex and different, and this makes it even more difficult to obtain their therapeutic inhibition in a precise way 9.

Because of the relevance of MMPs as regulatory enzymes in health and disease, it is important to understand how they are regulated in different conditions. In general, inducible MMPs are regulated at the transcriptional level by growth factors, chemokines, cytokines and ROS, and also by dietary molecules 10,11. Subsequent post-translational modification of the inactive zymogen (pro-enzyme) is obtained by removal of the pro-peptide domain to expose the active catalytic site (active enzyme). Following activation, MMPs are modulated by physiological inhibitors, *i.e*. the tissue inhibitors of matrix metalloproteinases (TIMPs) and α2 macroglobulin in plasma 12,13. In the course of MS, up-regulation of inducible MMP-9/gelatinase B increases the permeability of the BBB, facilitates the infiltration of leucocytes into the CNS, degrades the myelin sheath (*e.g*. myelin basic protein), and causes neuronal damage 3,7,8,14–16. On these grounds, elevation of MMP-9 in serum and CSF has been considered a biomarker of disease activity 17–20 and a therapeutic target for MS 7,21–23.

However, the role of MMP-9 in MS is complex: it involves different forms, multiple locations, different producer cell types, several mechanisms of intervention at different stages of disease, and different substrate specificities. For example, MMP-9 might have opposite effects on the disease. It degrades myelin constituents (directly or indirectly) in MS pathogenesis in the early stage of the disease, and contributes to remyelination in later stages, as oligodendrocytes might use MMP-9 to facilitate membrane process extensions 24.

Different forms of MMP-9 have been described, for instance, the inactive zymogen pre–pro-enzyme containing the signal peptide 25,26, and the pro-enzyme pro-MMP-9 of 92 kD. Serum pro-MMP-9 may be found covalently associated with NGAL (Neutrophil Gelatinase–Associated Lipocalin, or Lipocalin-2 [25 kD]). The activation of pro-MMP-9, mainly by MMP-3 27, occurs by removal of the N-terminal pro-peptide, yielding the 82 kD MMP-9 that is inhibited by its specific natural inhibitor TIMP-1. Carboxyterminal cleavage yields the 65 kD MMP-9, which is not inhibited by TIMP-1 28–31. Because of its molecular weight, very close to that of MMP-2, the 65 kD MMP-9 may be confused with an activated form of MMP-2 of about 68 kD. Finally, multimeric forms of MMP-9 have been described 32,33.

The finding that MMP-9, as other MMPs, also cleaves intracellular substrates, and shows different responsiveness to inflammatory molecules and therapeutic inhibitors depending on cell type and location, greatly complicates our understanding of the roles of MMP-9 in MS. For instance, it is not excluded that the cleavage of intracellular substrates, such as crystallins, contributes to the pathogenesis of MS 34–36. The accessibility of intracellular substrates to principally extracellular proteolysis may occur when cells succumb by apoptosis, necrosis or any other type of cellular disintegration 37. The analytical methods presently available for detecting gelatinases can describe only some of their specific properties, *i.e*. either gelatinase mRNA, or protein levels, expression and activity of gelatinases at the cellular level, but not one of the commonly used methods allows to distinguish post-translational modifications of the gelatinase isoforms (the charged variants in this article). Monodimensional (1-D) zymography does not provide information regarding the gelatinase variants produced by post-translational modifications. For example, classical zymography on gelatin-embedded gels reveals both the pro-form (the latent zymogenic form) and activation forms of gelatinases and shows the molecular mass diversity of their isoforms, but does not show any diversity if their molecular mass is similar, as may be the case for the 65 kD MMP-9 variant and the activation forms of MMP-2 38. It is known that MMP-9 can be either glycosylated, sialylated or nitrosylated, whereas MMP-2 may be phosphorylated 38–40.

Detection of gelatinase variants – *i.e*. the post-translationally modified isoforms of gelatinases – is essential to merge a more complete scenario of gelatinases in the course of MS.

At present, some information on gelatinase variants can be obtained by bidimensional (2-D) gel electrophoresis and proteomic analysis, but this approach is useful only when proteins collected from biological fluids are purified and concentrated before the application to the gel 41.

Here, we have refined the zymographic methodology to analyse the gelatinases present in the sera of patients with RR-MS by using 2-D zymography (2-DZ). Bidimensional zymography, which is based on isoelectrofocusing (1st dimension) and SDS gel electrophoresis on gels copolymerized with gelatin (2nd dimension), offers a more complete pattern of gelatinolytic enzymes, which are separated on the basis of their different isoelectric points (pI) and apparent molecular weights (Mw).

## Materials and methods

### Patients

Sera from 10 healthy controls (HC; six females, four males) and 33 patients with relapsing-remitting MS (RR-MS; 21 females, 12 males). All patients – 22 with RR-MS in remission phase (inactive RR-MS), 11 in relapse phase (active RR-MS) – were from Southern Italy. Patients were judged to have active disease according to clinical criteria and radiological evidence of some exacerbation, appearance of a new symptom or worsening of an existing symptom attributable to MS, accompanied by an appropriate new neurologic abnormality. All sera were obtained from the Hospitals ‘San Carlo’ (Potenza, Italy) and ‘Madonna delle Grazie’ (Matera, Italy) following the approval of the ‘Ethics Committee for Experimentation’ in Potenza (Resolution No. 132, 29 January 2008). Patients gave their informed consent and were treated independently from this study. All zymographic analyses shown in this paper were from RR-MS sera of patients not subjected to therapy.

### Detection of gelatinases by 1-D (1-DZ) and 2-D gelatin zymography (2-DZ)

Gelatinases present in the sera from MS patients or from HC were detected by SDS-PAGE gelatin zymography. Monodimensional scanning densitometry was used on an Image Master 1D program (Pharmacia Biotech, Uppsala, Sweden) platform. Levels of different gelatinase isoforms were expressed as optical density (OD) × mm^2^.

Gelatinase isoforms and charge variants present in the sera from MS patients or from HC were detected by 2-D gelatin zymography 42. Specifically, samples were applied under non-reducing conditions. Aliquots of 25 or 35 μl of sera were mixed with the rehydration solution containing 7 M urea, 2 M thiourea, 2% CHAPS (w/v), 0.5% (v/v) immobilized pH gradient (IPG) buffer, plus a trace of bromophenol blue, to a final volume of 250 μl. Isoelectrofocusing (IEF) was performed on IPG Dry-Strips of 13 cm with a linear pH gradient of four to seven or with a non-linear pH gradient of three to 11 (GE-Healthcare, Uppsala, Sweden). Immobilized pH gradient Dry-Strips were rehydrated with a sample-containing rehydration solution for 12 hrs at 20°C. Isoelectrofocusing was run by using an IPGphor unit (Amersham Biosciences, Uppsala, Sweden) at 20°C for a total of 36450 Vh. After IEF, IPG-strips were equilibrated for 20 min. by gentle shaking in equilibration buffer: 6 M urea, 30% (w/v) glycerol, 2% (w/v) SDS, 50 mM Tris-HCl (pH 8.8). In the second dimension, proteins were separated in a 7.5% or 8.5% (w/v) polyacrylamide gel copolymerized with 0.1% (w/v) gelatin on the SE 600 Hoefer System at 4°C first for 30 min. at 180 V and then for 5 hrs at 240 V. HMW-SDS Marker kits (Amersham Biosciences) or Precision Plus, Protein Standards Dual Color (Bio-Rad, Segrate, Italy) were used as molecular weight markers. After electrophoresis, for the development of enzymatic activities, gels were washed two times in washing buffer and then incubated for 14 hrs at 37°C in developing buffer. Gels were then stained with Coomassie Brilliant Blue R-250 (for 30 min. at room temperature) and destained in methanol/acetic acid/H_2_O. The 2-DZ gels were scanned by using a ScanMaker 9800 XL-Microtek (Hsinchu, Taiwan). The images were digitally converted from positive to negative images. Image analysis for the determination of apparent Mw and pI was carried out by using the ImageMaster 2D Elite V. 2002.01 software (Amersham Biosciences) and enzyme levels were expressed as Spot Volume (corresponding to the sum of pixel intensities within the digested spot). The individual digested spot volumes were normalized as a percentage of the total volume of all digested spots present in the gel.

### Purification of gelatinases by gelatin affinity chromatography

Gelatinase purification was performed by miniature gelatin affinity chromatography 43. Briefly, 0.5 ml of pooled RR-MS sera, diluted with 0.5 ml of 50 mM Tris pH 7.5, 0.6 M NaCl (equilibration buffer), were added to mini-spin columns (Bio-Rad Laboratories) loaded with 0.5 ml gelatin-Sepharose 4B beads (GE Healthcare, Uppsala, Sweden; 1/3 beads in 2/3 equilibration buffer) previously equilibrated with two volumes of equilibration buffer. The mixture was then equilibrated at 4°C for 60 min. (vortexing every 10 min. and reversing the column).

Successively, the cap was removed and the unbound sample was removed by centrifugation at 3000 × *g* (Amicon MC-13 microcentrifuge; Millipore, Bedford, MA, USA), for 2 min. Beads were washed two times with washing buffer 50 mM Tris pH 7.5, 0.1 M NaCl, and gelatinases were eluted with 150 μl of 5% DMSO in 50 mM Tris pH 7.5, 0.1 M NaCl after incubation for 30 min. at room temperature. The eluate was totally recovered by centrifugation at 2000 rpm for 2 min., and then desalted and concentrated to a final volume of 30 μl with 2.0 ml of water in Vivaspin 500 (MWCO 30,000; GE Healthcare). Gelatinase levels present in the purified fraction (corresponding to the pooled RR-MS sera) were analysed by 2-D gelatin zymography.

### Desialylation

To 20 μl of purified gelatinases obtained from 10 pooled RR-MS sera, were added 5 μl of reaction buffer and 5 μl (0.5 U/μl) of α(2-3,6,8,9) neuraminidase from *Arthrobacter ureafaciens* (N 3786; Sigma-Aldrich, Sigma, St. Louis, MO, USA). Digestions were performed at 37°C for 6 hrs. The samples were then diluted with 220 μl of the rehydrating solution of the IPG strips and the desialylated fraction was analysed by 2-DZ as described above.

### Western blot analysis

After electrophoretic (E) runs 1-DE or 2-DE, gels without gelatin substrate were rinsed in transfer buffer and blotted on a nitrocellulose membrane (for 90 min. at 350 mA), using a MINI-trans Blot apparatus (Bio-Rad Laboratories). After transfer, the membranes were incubated with primary antibodies: Anti-MMP-9 (Ab-8) Mouse mAb (IA5) at concentration of 2.66 μg/ml (recognizes the 92 kD latent and the 86 kD active forms), Anti-MMP-2 (Ab-4) Mouse mAb (75-7F7) at concentration of 1.0 μg/ml (Calbiochem, Milano, Italy; recognizes the 72 kD latent and the 66 kD active forms), Anti-MMP-9 (4A3) at concentration of 2.0 μg/ml (Novus Biologicals, Novus Biologicals, Segrate, MI, Italy; recognizes the 82 kD and the 65 kD active forms) and Anti-NGAL Antibody (5G5) at concentration of 2.0 μg/ml (EuroClone, Pero, MI, Italy; recognizes the human NGAL). Thereafter, the membranes were incubated with the HRP-labelled secondary antibody (anti-mouse, Invitrogen, San Giuliano Milanese, MI, Italy or Sigma-Aldrich). Finally, detection was carried out with ECL reagent (GE Healthcare) for detection on X-ray film (Fuji RX). Images were acquired by using a ScanMaker 9800 XL-Microtek.

### Concanavalin A chromatography

To distinguish the 65 kD MMP-9 from of MMP-2, RR-MS serum, concentrated by affinity chromatography on gelatin-Sepharose, was purified by concanavalin-A-Sepharose affinity chromatography (Sigma-Aldrich) according to the manufacturer with minor modifications.

Briefly, a mini-spin column with 500 μl of concanavalin-A-Sepharose was pre-washed with five column volumes of wash solution (1 M NaCl, 5 mM MgCl_2_, 5 mM MnCl_2_, 5 mM CaCl_2_) and equilibrated with 20 mM Tris, pH 7.4, 0.5 M NaCl (equilibration buffer).

A volume of 75 μl of concentrated RR-MS sera, diluted with 225 μl of equilibration buffer, was added to mini-spin columns, the mixture was then incubated at 4°C for 60 min. (vortexing every 10 min. and reversing the column). After sample application, the unbound fraction was recovered by centrifugation (pass through) and the column was washed first with 300 μl of equilibration buffer and then with 50 mM methyl α-D-mannopyranoside in equilibration buffer. Finally, the column was eluted with the equilibration buffer containing 0.5 M methyl α-D-mannopyranoside. Successively, the fractions were desalted and concentrated to a final volume of 30 μl with 2.0 ml of water in Vivaspin 500 (MWCO 10,000; GE Healthcare) and then analysed by gelatin 2-D zymography.

## Results

### Analysis of serum samples by 1-D zymography (1-DZ)

**Fig 1:**
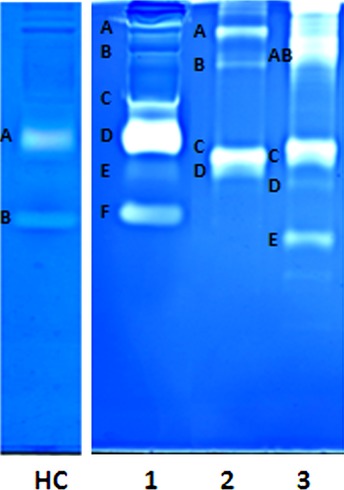
Monodimensional zymography (1-DZ) on 7.5% polyacrylamide containing 0.1% gelatin. Lane HC: 2 μl of serum from healthy control; Lane 1: 2 μl of serum from a patient with inactive RR-MS; Lane 2: 10 ng of recombinant human proMMP-9 produced in insect cells; Lane 3: 10 ng human recombinant pro-MMP-9 produced in CHO cells.

The proteolytic profile of serum samples from patients with relapsing-remitting (RR) MS was analysed by using classical mono-dimensional zymography in non-reducing conditions. A representative zymogram of a serum sample from a RR-MS patient is shown in Figure 1 (lane 1). Resulting bands were compared with two different MMP-9 standard preparations (lanes 2 and 3). The standard applied on lane 2 was an insect-derived human recombinant MMP-9 44, whereas the standard applied on lane 3 was a human recombinant MMP-9 produced in Chinese hamster ovary (CHO) cells (Calbiochem). The standard bands 2A, 2B and the broad band 3AB corresponded to the multimeric forms of MMP-9; bands 2C and 3C corresponded to pro-MMP-9; bands 2D and 3D corresponded to the activation forms of MMP-9; and finally the lower 3E band (present only in the Calbiochem Standard) of about 50 kD probably corresponded to an autolytic fragment.

When comparing the MMP-9 standard profiles shown in lanes 2 and 3 with the gelatinase profile present in the RR-MS serum (lane 1), we noticed that the main band of the standards and serum migrated with different mobilities. The band 1F of ∼68 kD corresponded to MMP-2. In this respect, it should be mentioned that 1-DZ is routinely applied for the analysis of gelatinases in biological fluids and the assignment of the zymographic bands to either gelatinases A or B has been confirmed many times.

Lane HC shows a HC serum. In healthy individuals, only pro-MMP-9 (band A) and MMP-2 (band B) are present. The MMP levels in HC sera were always lower than in MS sera, as can also be deduced from this figure. Additional bands are present only in some MS sera: in particular two bands around 170 and 120 kD (an example of the latter is the band 1C in Fig. 1) corresponding to the covalent MMP-9/NGAL complex.

### Identification of gelatinases by Western blot analysis

The identification of gelatinases by Western blot was first carried out by 1-D zymographic and electrophoretic analyses. In view of the fact that zymographic analysis has a sensitivity in the picogram range, whereas Western blot analysis has a sensitivity in the nanogram range, we concentrated the samples prior to Western blot analysis.

Fivefold concentrated (Vivaspin 500, MWCO 50,000; GE Healthcare) serum samples from either HC or RR-MS patients were subjected to 1-DZ and 1-DE. The latter were transferred on nitrocellulose membrane and treated with different antibodies, as described in the materials and methods section.

**Fig 2:**
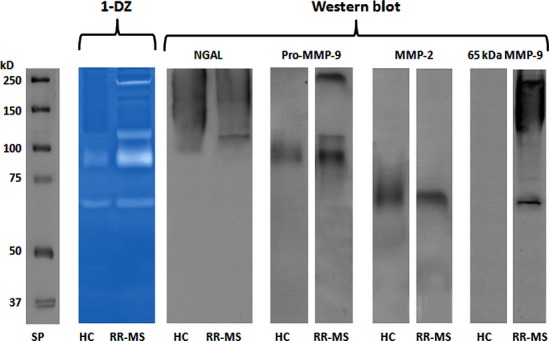
1-DZ analysis and identification of gelatinases by Western blot. 1.5 μl of serum from healthy control (HC) or from a patient with inactive RR-MS were loaded on a 7.5% polyacrylamide gel copolymerized with 0.1% (w/v) gelatin (1-DZ). For Western blot analysis, 2 μl of fivefold concentrated (Vivaspin 500, MWCO 50,000; GE Healthcare) serum samples (the same as in 1-DZ) were separated on a 7.5% polyacrylamide gel without gelatin (1-DE), transferred on nitrocellulose membranes and incubated with the following primary antibodies: Anti-NGAL Antibody (5G5) at concentration of 2.0 μg/ml; Anti-MMP-9 (Ab-8) Mouse mAb (IA5) at concentration of 2.66 μg/ml; Anti-MMP-2 (Ab-4) Mouse mAb (75-7F7) at concentration of 1.0 μg/ml and Anti-MMP-9 Ab (4A3) at concentration of 2.0 μg/ml. Standard Proteins (SP): Precision Plus Protein Standards Dual Color (Bio-Rad).

As shown in Figure 2, in the control (HC) sample, only the 92 kD pro-MMP-9 and the MMP-2 bands were detected, whereas, in the RR-MS sample, three additional bands were present, corresponding to the MMP-9 multimeric form, the NGAL-pro-MMP-9 complex, and the 65 kD MMP-9 form, respectively. The increase in brightness and importance of the 92 kD MMP-9 band in RR-MS in comparison with the control is a result of the appearance of a 4th band corresponding to the activated 82 kD MMP-9 form, that can hardly be distinguished in 1-DZ, but will become visible in 2-DZ.

### Analysis of serum samples by 2-D zymography and Western blot

1-DZ analysis provides important information on the presence of gelatinases in serum samples, but the nature of this information is limited because it is restricted to Mw differences. Post-translational isomeric forms with the same Mw might be detected only by applying an isoelectric focusing step (fractionation on the basis of different isoelectric points/pIs), as is the case with 2-DZ.

**Fig 3:**
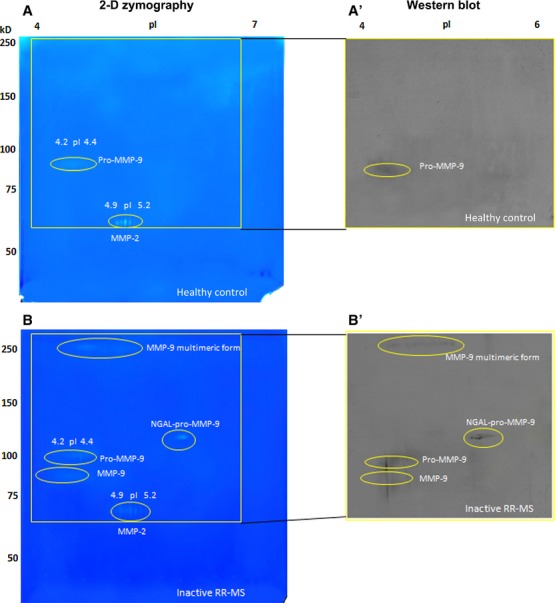
2-DZ analysis and identification of MMP-9 by Western blot. Left panels (A and B): 2-D zymography (2-DZ) of serum samples from healthy control (A) and from an inactive RR-MS patient not subjected to therapy (same patient as in Fig. 2; B). For 2-DZ, aliquots of 35 μl of serum were resuspended in the rehydration solution and subjected to isoelectrofocusing (IEF; 1st dimension) on IPG Dry-Strips of 13 cm in a linear pH gradient of 4–7. After IEF, IPG strips were equilibrated and then applied for the 2nd dimension in a 8.5% (w/v) polyacrylamide gel copolymerized with 0.1% (w/v) gelatin. The isoforms and charge variants of MMP-2 and MMP-9 appear as clear spots of digestion on the dark background of the gel. Right panels (A' and B') represent Western blot analysis of the same sera shown in A and B. Aliquots of 35 μl of serum samples (instead of the usual 20 μl) were subjected to 2D electrophoresis (2-DE) by using 8.5% (w/v) polyacrylamide gels without gelatin. After transfer of the proteins, the nitrocellulose membranes were incubated with an anti MMP-9 (Ab-8) Mouse mAb (IA5) at concentration of 2.66 μg/ml.

The 2-DZ profiles of gelatinases present in control (HC) and in RR-MS samples are shown in Figure 3 [panels A and B, respectively (on the left)]. On the right panels A' and B', we show the corresponding Western blot analyses, obtained after the transfer on nitrocellulose membranes from the 2-DE gel and after treatment with anti-pro-MMP-9 antibody.

The higher acrylamide percentage (8.5%) applied in this analysis allowed a better separation of pro-MMP-9 from the activated 82 kD MMP-9. From top to bottom, five groups of spots were detected: (*i* ) the multimeric form of pro-MMP-9 (Mw: <200; pI 4.2–4.5); (*ii* ) the NGAL-pro-MMP-9 complex (Mw 116 and pI range 5.6–5.9); (*iii* ) the pro-MMP-9, represented by a broad lysis zone around Mw 90–92; (*iv* ) the activated MMP-9 of about 80–82 kD, and pI 4.2–4.4; and finally, a group of spots corresponding to MMP-2 (Mr 68 kD and pI range 4.9–5.2). The detection of the 65 kD MMP-9 form observed in 1-DZ was not well detected in this gel and will be shown later in another gel.

### The charge variants of gelatinases

As shown for the first time by 2-DZ, all gelatinase isoforms consisted of a number of charge variants. Matrix metalloproteinases-2: 5-7 charge variants. Matrix metalloproteinases-9: four charge variants in the NGAL-pro-MMP-9 complex, six charge variants for the 65 kD MMP-9 (shown later), and several unresolved and undefined charge variants in the case of multimeric MMP-9, pro-MMP-9, and activated 82 kD MMP-9.

In the HC (panel A), only the pro-MMP-9 and MMP-2 forms were detected, whereas the lytic spots corresponding to the multimeric form of pro-MMP-9, the NGAL-pro-MMP-9 complex and the MMP-9 activated forms were not present.

Western blot analyses, carried out with anti-pro-MMP-9 antibody (right panels), were in agreement with those observed in 1-DZ analysis (Fig. 2). Western blot with anti-NGAL, anti-MMP-2 and anti-65 kD MMP-9 will be shown in forthcoming gel examples.

### Are the charge variants of MMPs due to post-translational modifications?

#### Detection of sialylated MMP-9 forms and desialylation

To detect whether the broad lysis areas in the acidic region between 4.2 and 4.4 can be ascribed to the presence of sialic acid, serum samples from four different patients with RR-MS were enriched and concentrated by affinity chromatography on gelatin-Sepharose, as described in the materials and methods section.

The detection of the sialylated forms of MMP-9 was made easier after concentration of the sera ten times. By contrast, in HC, this broad lysis area in the acidic region was observed only if the samples were concentrated 40 times, a result indicating that the acidic MMP-9 may be present also in healthy individuals, but at much lower levels than in RR-MS.

**Fig 4:**
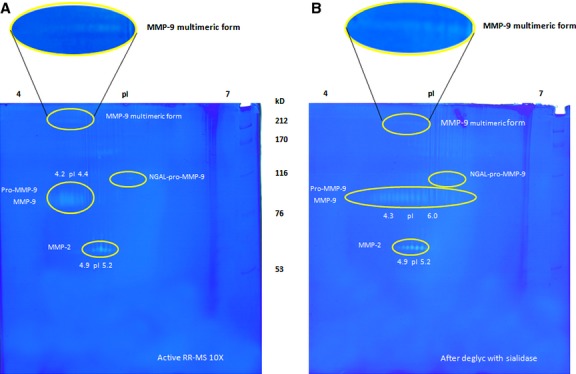
Detection of sialylated MMP-9 isoforms by 2-DZ and desialylation with neuraminidase. (A) 2-D zymography (2-DZ) of 20 μl of a pool of sera obtained from four active RR-MS patients, after enrichment (10 × ) by gelatin-Sepharose affinity chromatography. (B) Analysis by 2-DZ of the different MMP-9 isoforms after desialylation of 20 μl of the enriched (10 × ) pool of MS sera with neuraminidase (0.5 U/μl) for 16 hrs at 37°C. Sialylated forms of MMP-9 were detected for the acidic forms and the multimeric forms of MMP-9, but not for NGAL-pro-MMP-9. The oval box on the top shows a magnification of the multimeric forms of MMP-9.

To demonstrate that the acidic forms of MMP-9 in the acidic region were sialylated, 20 μl of RR-MS sera, enriched by gelatin-Sepharose affinity chromatography, was subjected to desialylation by incubation with neuraminidase. As shown in Figure 4B, treatment with neuraminidase shifted the MMP-9 spots from pI 4.2–4.4 to pI 4.4–6.0. A similar shift of the multimeric forms of MMP-9 towards the more basic region suggests that these are sialylated too. However, the pIs of the multimeric forms varied slightly in different sera (data not shown).

#### Demonstration of MMP-2 phosphorylation

The pIs and relative levels of the five MMP-2 spots were determined and are shown in Table 1. In some cases, the number of detected MMP-2 spots increased to seven, but the central spot always remained the most abundant one. As the position of charge variants of MMP-2 did not change in desialylation experiments, we evaluated whether MMP-2 spots in clinical samples refer to different degrees of phosphorylation.

**Table 1:** 2-DZ analysis of MMP-2 and effect of treatment with phosphatase

		pI	
Spot	Relative zymolytic level	Without phosphatase	After phosphatase treatment
1	18	5.005	5.129
2	13	5.048	5.175
3	52	5.110	5.228
4	13	5.169	5.276
5	5	5.226	5.330

RR-MS pooled sera, enriched in gelatinases by affinity chromatography on gelatine-Sepharose, were incubated (O/N, at 37°C) with alkaline phosphatase (Sigma-Aldrich), and then analysed by 2-DZ. The isoelectric point of the digested spots was measured by the ImageMaster2D Elite V. 2002.01 software (Amersham Biosciences).

A volume of 20 μl of RR-MS pooled sera, enriched in gelatinases by affinity chromatography on gelatin-Sepharose, was incubated (overnight at 37°C) with alkaline phosphatase, and then analysed by 2-DZ. The zymographic patterns of the samples treated with phosphatase were similar to those of untreated sera (control), except for the pI values of the five MMP-2 spots, which exhibited pI values more basic than those in the untreated control samples. The alterations of the pIs were in line with partial dephosphorylation.

#### Identification of NGAL-MMP-9 complex, MMP-2 and 65 kD MMP-9 by Western blot analysis

**Fig 5:**
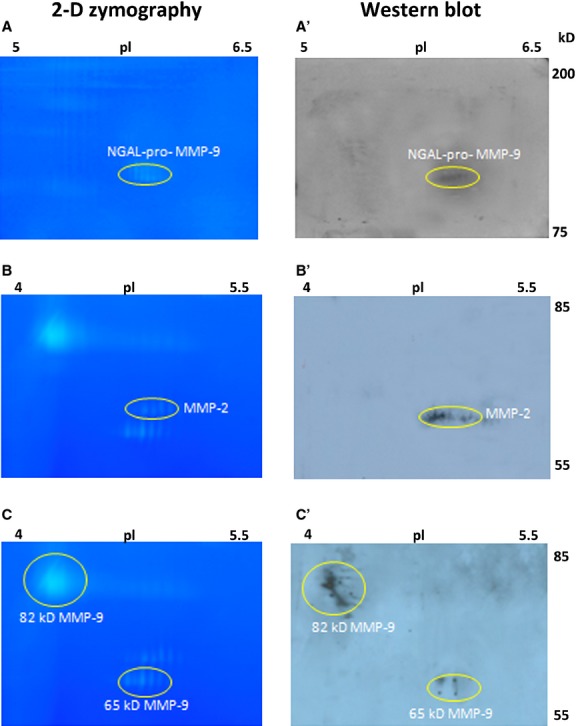
Identification of gelatinases by Western blot analysis. A pool of sera from 10 patients with inactive RR-MS was used. For 2-DZ, pooled sera were concentrated 10-fold and 40 μg of total proteins were applied (left panels). For Western blot analysis pooled sera were concentrated 40-fold and 160 μg of total proteins were subjected to 2-DE (right panels). 2-DE gels were cut and the corresponding nitrocellulose membranes were treated with anti-NGAL Ab (A'), anti-MMP-2 Ab (B') and Anti-MMP-9 (4A3) Ab, recognizing the 65 kD and the 82 kD activated forms (C'). Gel A shows the region with isoelectric point between pH 5–6.5 and molecular mass between 200 and 75 kD, analysed by Western blot in A'; Gels B and C show the region with isoelectric point between pH 4–5.5 and molecular mass between 85 and 55 kD, analysed by Western blot in B' and C', respectively.

The identification of gelatinases was carried out in a 10-fold concentrated serum sample from RR-MS patients. The concentrated pool (40 μg) was subjected to 2-DZ (Fig. 5, left panels), whereas the corresponding membranes from the 2-DE gel, were cut and treated with anti-NGAL Ab (panel A'), anti-MMP-2 (panel B') and anti-65 kD MMP-9 4A3 Ab (panel C'), respectively (Fig. 5, right panels). The membrane treatment with an anti-NGAL antibody was in line with the zymographic observation of NGAL-MMP-9 complex in MS sera (Fig. 3B). As shown in Fig. 5C', the anti-MMP-9 4A3 Ab recognized the two activated forms of 65 kD and 82 kD of MMP-9.

#### The 65 kD activation form of MMP-9

It is known that MMP-9, in addition to the activated 82 kD form, has a 65 kD truncation form. The 65 kD MMP-9 has been relatively neglected, because 1-DZ and ELISA do not allow distinguishing it from MMP-2 and other MMP-9 forms, respectively. In particular, in 1-DZ the 65 kD MMP-9 and the 66–68 kD MMP-2 migrate at similar positions. Only recently, the two enzymes were distinguished by using ConA Sepharose to separate MMP-2 from the 65 kD MMP-9 on the basis of their different glycosylation 30.

**Fig 6:**
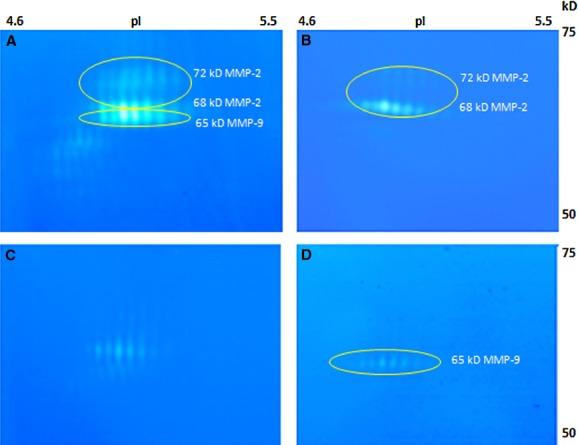
2-DZ analysis of gelatinase forms before and after concanavalin-A-Sepharose chromatography. 75 μl of a 10 ×  concentrated pool of sera from two patients with active RR-MS were subjected to concanavalin-A-Sepharose chromatography as described in the methods. Fractions obtained were desalted and concentrated and then analysed by 2-D zymography. 25 μl of samples were loaded on the gels. (A) concentrated pool before concanavalin-A-Sepharose chromatography; (B) unbound fraction (‘pass through’) corresponding to MMP-2; (C) wash step, with residual MMP-2; (D) eluted fraction from concanavalin-A-Sepharose chromatography.

As shown in Figure 6 by 2-DZ, concentration on gelatin-Sepharose of serum samples made it possible to detect, in addition to MMP-2, an additional series of spots, which were assigned to the 65 kD MMP-9 (Fig. 6A). Chromatography on ConA Sepharose confirmed the co-presence of both enzymes in the lower region of the gel. Panel B shows the zymographic profile of the pass through (non-adsorbed fraction), corresponding to MMP-2. Panel C is related to the wash steps. Panel D corresponds to the eluted fraction, shown by Western blot analysis to be the 65 kD MMP-9 (Fig. 5C, left panel).

## Discussion

Matrix metalloproteinases-9 and MMP-2 display a complex variety of functions in health and disease and this implies also that their regulation might be complex 45. Structural complexity is related to oligo-and heteromerization with NGAL, post-translational processing named activation and truncation, glycosylation (*e.g*. sialylation) and phosphorylation. We found that all isoforms of both gelatinases exist in the form of multiple charge variants, some of which were based on sialylation (MMP-9) or phosphorylation (MMP-2).

A summary of the complete gelatinase pattern observed in this study is shown in Table 2. Because of this intrinsic complexity, a simple assessment of MMPs levels in body fluids is no more sufficient to understand their role in diseases. When restricting the attention to MMP-9, assumed to be the most important MMP immune effector implicated in MS pathogenesis, it is possible to note that it is secreted as inactive forms (the zymogen pro-MMP-9 multimers and monomers and pro-MMP-9 associated with lipocalin) and its activity is related to at least two activation forms (the N-terminal truncated 82 kD form; the N-and C-terminal truncated 65 kD form).

**Table 2:** Total pattern of different forms of gelatinases detected in MS sera

Gelatinase isoforms		Charge variants	pI	Mw
Gelatinase B (MMP-9)	Multimeric form	Several (unresolved)	4.2–4.5	>200
	NGAL-pro-MMP-9 complex	1	5.61	116
		2	5.72	116
		3	5.80	116
		4	5.92	116
	pro-MMP-9	Several (unresolved)	4.1–4.6	90–92
	82 kD MMP-9	Several (unresolved)	4.1–4.6	80–82
	65 kD MMP-9	1	4.82	65
		2	4.86	65
		3	4.92	65
		4	4.99	65
		5	5.06	65
		6	5.15	65
Gelatinase A (MMP-2)	MMP-2	1	4.97	68
		2	5.03	68
		3	5.09	68
		4	5.16	68
		5	5.21	68

Image analysis of 2-DZ was carried out by the ImageMaster2D Elite V. 2002.01 software (Amersham Biosciences).

The activated isoforms probably have different roles, are more or less sensitive to endogenous inhibitors and are differently prone to post-translational modifications. Although it is known that MMP-9 can be either glycosylated, sialylated or nitrosylated, whereas MMP-2 may be phosphorylated, the role of these post-translational modifications has not been ascertained yet, in particular in MS. This may be ascribed also to the fact that until now it was technically not possible to investigate in detail the structural changes that may occur in MMPs with the limited amounts of clinical samples and the available techniques. The charge variants, described here, conceivably may bind with different affinities to substrates, receptors, inhibitors and activating enzymes.

Current methods for detecting MMP mRNAs or proteins are numerous: monodimensional gelatin zymography (1-DZ); *in situ* gelatin zymography; Western blotting; *in situ* hybridization; quantitative PCR; immunohistochemistry; enzyme-linked immunoassays; and enzyme activity assays (*e.g*., using fluorogenic peptide-, radiolabeled-, and fluorescent dye-linked substrates). With the use of these approaches, MMPs can be described by activity, mRNA, protein levels, and expression. However, not one of these techniques can provide direct information on post-translational modifications of the enzymes and charge variants with similar molecular masses.

With the use of 2-DZ, it is possible to detect the presence of charge variants and post-translational modifications leading to micro-heterogeneity. We revealed, for the first time, the different isoforms and charge variants of gelatinases present in the sera of MS patients and HC. Four variants of N-GAL-pro-MMP-9, six of the 65 kD MMP-9, and several undefined charge variants for the MMP-9 isoforms were detected, whereas five to seven variants of MMP-2 were observed. As assessed by 2-D zymography of recombinant MMP-9 or of human control sera, the presence of charge variants is not restricted to MS patients and appears to be characteristic of both MMP-9 and MMP-2. The numbers of variants and their apparent relative ratios do not change in the pro-forms or in the activation forms. As shown in http://www.nextprot.org there are 20 variants of MMP-9, some of these with different pI of the protein.

The second finding reported in this paper is that the multimeric MMP-9, the pro-MMP-9 and the 82 kD activation form of MMP-9 are sialylated. Glycan analysis of leucocyte-derived MMP-9 was previously used to show the presence of core-fucosylated and sialylated oligosaccharides in human MMP-9 46.

In the course of MS relapses, all zymolytic MMP-9 spots increased in intensity and new diffuse spots appeared in the acidic region of the gel, corresponding to sialylated MMP-9. The increase in sialylated 82 kD MMP-9 activation form during relapses is because of its truncation, but also suggests a regulatory role of glycosylation. With regard to the other activated forms of MMP-9, the 65 kD MMP-9 does not appear to be sialylated. Sialylation of MMP-9 has been shown to alter its interaction with TIMP-1 47.

Serum proteomic analysis has been suggested as an analytical tool for MS, but has also been found extremely complex in the biomarker field. With the present method, we start from a marker, associated with MS (*e.g*. MMP-9) and add information about post-translational modifications (that also may be associated with MS). In this way, the information content of MMP-9 as a biomarker for MS may be reinforced. For instance, further studies should clarify the causes of the presence of multiple charge variants of gelatinases. At present, as these enzymes contain a large number of arginines, our working hypothesis is that, aside protein micro-heterogeneity, the enzymes may be differentially citrullinated.

It is critical to notice that the serum alterations, observed in this study, may be the reflection of local alterations of MMPs in the CNS and be based on changes of MMPs during demyelination or (re)myelination processes. For example, the relevance of such activities was expressed in recent publications about myelin-forming and altering Schwann cell 48,49. However, we do not exclude other possible explanations, such as induction of differentially modified MMP forms in the liver during inflammations.

Altogether, this work stimulates further investigations into the roles of MMP isoforms and charge variants in MS, for a better understanding of MS pathogenesis, a better evaluation of the response to therapy and the set up of better diagnostic procedures.

## Conclusions

In this work, the intrinsic structural complexity of the metalloproteinases MMP-2 and MMP-9 is highlighted. The use of 2-DZ showed not only the already known MMP isoforms (the pro-forms and the activation forms of monomers and multimers) but revealed also, for the first time, a number of charge variants in all isoforms of both enzymes. In addition, activated 82 kD MMP-9 forms were shown to be sialylated, whereas activated MMP-2 appeared to be phosphorylated. All isoforms and charge variants were observed in concentrated serum samples without the need to purify the enzymes to homogeneity. The structural complexity of MMPs is probably in line with their numerous functions in physiopathology and must be taken into account in addition to their regulation by their natural inhibitors TIMPs.

With regard to MS, the aim of this study was to find and to describe all possible isoforms and isomers (the charge variants with different pIs, in this study) that can be found in the sera of MS patients.

It was not yet our intention to find any correlation with the course of the disease or to evaluate the effectiveness of therapy on MMP isoform levels. However, with a focus on MMP-9 activated isoforms, we have found that the C-terminal truncated 65 kD MMP-9 appears only if the sialylated 82 kD MMP-9 is present and only in MS patients, whereas the opposite was not observed. These data suggest that the formation of the truncated 65 kD MMP-9 form is related to an inflammatory process. This is also in accordance with knowledge about glycosylation as post-translational modification of proteins 50 and with the observation that the 65 kD MMP-9 is not inhibited by TIMPs, an aspect that well correlates with a pathogenetic role.

It is important to note that the 65 kD form cannot be distinguished from MMP-2 with classical 1-DZ, whereas this distinction is possible with the new technique. It may be concluded that because of their intrinsic complexity, a simple assessment of MMPs levels in body fluids is no more sufficient to understand their role in disease pathogenesis.
